# Toxicity of particles derived from combustion of Ethiopian traditional biomass fuels in human bronchial and macrophage-like cells

**DOI:** 10.1007/s00204-024-03692-8

**Published:** 2024-03-01

**Authors:** Sarah McCarrick, Mathilde N. Delaval, Ulrike M. Dauter, Annette M. Krais, Anastasiia Snigireva, Asmamaw Abera, Karin Broberg, Axel C. Eriksson, Christina Isaxon, Anda R. Gliga

**Affiliations:** 1 Institute of Environmental Medicine, Karolinska Institutet, Stockholm, Sweden; 2 Joint Mass Spectrometry Centre (JMSC), Cooperation Group Comprehensive Molecular Analytics, Helmholtz Munich, Neuherberg, Germany; 3 Division of Occupational and Environmental Medicine, Department of Laboratory Medicine, Lund University, Lund, Sweden; 4 Ergonomics and Aerosol Technology, Lund University, Lund, Sweden; 5 NanoLund, Lund University, Lund, Sweden; 6 Addis Ababa University, Addis Ababa, Ethiopia

**Keywords:** Solid biomass fuel, Combustion-derived particles, Toxicity, Genotoxicity, Cytokines, Polycyclic aromatic hydrocarbons (PAH)

## Abstract

The combustion of traditional fuels in low-income countries, including those in sub-Saharan Africa, leads to extensive indoor particle exposure. Yet, the related health consequences in this context are understudied. This study aimed to evaluate the in vitro toxicity of combustion-derived particles relevant for Sub-Saharan household environments. Particles (< 2.5 µm) were collected using a high-volume sampler during combustion of traditional Ethiopian biomass fuels: cow dung, eucalyptus wood and eucalyptus charcoal. Diesel exhaust particles (DEP, *NIST* 2975) served as reference particles. The highest levels of particle-bound polycyclic aromatic hydrocarbons (PAHs) were found in wood (3219 ng/mg), followed by dung (618 ng/mg), charcoal (136 ng/mg) and DEP (118 ng/mg) (GC–MS). BEAS-2B bronchial epithelial cells and THP-1 derived macrophages were exposed to particle suspensions (1–150 µg/mL) for 24 h. All particles induced concentration-dependent genotoxicity (comet assay) but no pro-inflammatory cytokine release in epithelial cells, whereas dung and wood particles also induced concentration-dependent cytotoxicity (Alamar Blue). Only wood particles induced concentration-dependent cytotoxicity and genotoxicity in macrophage-like cells, while dung particles were unique at increasing secretion of pro-inflammatory cytokines (IL-6, IL-8, TNF-α). In summary, particles derived from combustion of less energy dense fuels like dung and wood had a higher PAH content and were more cytotoxic in epithelial cells. In addition, the least energy dense and cheapest fuel, dung, also induced pro-inflammatory effects in macrophage-like cells. These findings highlight the influence of fuel type on the toxic profile of the emitted particles and warrant further research to understand and mitigate health effects of indoor air pollution.

**Supplementary Information:**

The online version contains supplementary material available at 10.1007/s00204-024-03692-8.

## Introduction

Air pollution poses a threat to human health worldwide (Landrigan et al. 2018) and is the second leading risk factor for death in Africa, being responsible for 1.1 million deaths in 2019 (Fisher et al. 2021). The African continent is facing the double burden of both high indoor and high ambient outdoor air pollution, and more than 60% of air-pollution related deaths were linked to household air pollution (HAP) in 2019 (Fisher et al. 2021). Worldwide, it is estimated that three billion people are exposed to HAP generated from combustion of solid fuels mainly during cooking and heating, as solid fuels are still the primary energy supply for many households, particularly in rural areas of low-income countries (WHO 2014). In sub-Saharan Africa, approximately 75% of the population rely on burning of traditional solid fuels (Health Effects Institute 2022).

The most important determinant of the choice of fuel is its cost as depicted in the so-called energy ladder (Sood 2012). Solid fuels such as animal dung and crop residues are placed lowest in the energy ladder and are associated with a low socioeconomic status, followed by wood and charcoal (Sood 2012). Generally, these fuels have poor energy production efficiency and generate more emissions compared to cleaner fuels, such as gas. The incomplete combustion of solid fuels on an open fire or traditional stove generates high levels of hazardous pollutants including particulate matter (PM), polycyclic aromatic hydrocarbons (PAHs) and volatile organic compounds (VOCs) (WHO 2016). Indoor air pollution from traditional biomass fuels such as dung, wood or charcoal has been associated with an increase in the incidence of respiratory infections and chronic respiratory diseases, cardiovascular diseases, and all-cause mortality both in adults and children (Po et al. 2011, Kodgule et al. 2012, Kurmi et al. 2012, Balmes 2019). Women and children bear the biggest health burden due to their household roles resulting in more extensive exposure (Misra et al. 2018; Okello et al. 2018; Adane et al. 2020; Andualem et al. 2020; WHO 2022).

The International Agency for Research on Cancer (IARC) has classified the use of coal in household settings as Group 1 (carcinogenic) and use of biomass fuels as Group 2A (probably carcinogenic) (IARC 2010). The classification was based on limited mechanistic data and our understanding of the toxicity of HAP emissions generated from combustion of solid biomass fuel as well as toxic mechanisms is relatively incomplete. Overall, characteristics and toxicity of combustion products seems to be dependent on the type of fuel (including the different sources of wood), types of stove and combustion conditions. Experimental studies in rodents indicated acute neutrophilic responses, followed by sub-chronic eosinophilic and lymphocytic responses after exposure to PM collected from rural Indian homes, where particles derived from dung were more potent under acute conditions compared to wood particles, but vice versa under sub-chronic conditions (Sussan et al. 2014). A study in mice further showed a development of acute lung injury following exposure to emissions from the combustion of red oak wood on traditional cookstoves (Gibbs-Flournoy et al. 2018). In vitro studies reveal that particles derived from the combustion of various solid fuels, including different types of wood and dung, can cause inflammatory activity, cell death and DNA damage (Tapanainen et al. 2012; Uski et al. 2014; McCarthy et al. 2016, 2017). Particles from animal dung combustion have been shown to possess a considerable oxidative activity (Mudway et al. 2005). Cellular toxic effects caused by PM derived from wood combustion, including oxidative stress and genotoxicity, have been suggested to be related to the chemical composition of the PM including PAHs (Tapanainen et al. 2012; Arif et al. 2017; Krapf et al. 2017; Marabini et al. 2017; Kim et al. 2018).

Despite some understanding of the toxicity of HAP in general, there are considerable knowledge gaps for HAP in the sub-Saharan region. Notwithstanding the high exposure with average indoor levels of Particulate Matter 2.5 (PM_2.5_) in Ethiopia of 477 μg/m^3^ (Asefa et al*.* 2022), the negative health effects of HAP are understudied in this region (Katoto et al. 2019; Isaxon et al. 2022). There is a need for improved understanding of the toxicity and underlying mechanisms of HAP using relevant traditional fuels and realistic combustion conditions. The aim of this study was to evaluate and compare the toxicity of well-characterized combustion particles derived from Ethiopian traditional biomass fuels with different energy densities, such as eucalyptus wood, eucalyptus charcoal and cow dung, which are relevant for indoor sub-Saharan environments. For this purpose, bronchial epithelial cells (BEAS-2B) and monocyte derived macrophages (THP-1) were used as in vitro models to measure cytotoxicity, inflammation, and genotoxicity after acute exposure to combustion-derived particles from traditional Ethiopian biomass fuels.

## Materials and methods

### Solid biomass fuels, combustion, and particle collection

Biomass fuels including charcoal made from eucalyptus, wood of eucalyptus and dried cow dung were brought from central Ethiopia to Lund University, Sweden and used without modification. Combustion particles from solid fuels were generated as previously described in Eriksson et al. (2022). Briefly, the combustion of solid fuels was performed in a laboratory setup but under conditions and with procedures representative for real-world settings in low-income countries. A traditional Ethiopian stove for cooking was used for the combustion of charcoal whilst a 3-stone setup was used for the remaining fuels. The combustion took place in a 1.33 m^3^ stainless steel chamber and the aerosol created was captured by an extractor hood placed above the fire. Using an air amplifier (Coval M10C, Releigh, USA), the air was further transported by a flow of clean air into a 21.6 m^3^ stainless steel chamber, with an air exchange rate of 15 h^−1^ where the particle collection and aerosol characterization was conducted, as previously described in Isaxon et al*.* (2013). Particles < 2.5 µm were collected on Polytetrafluorethylene (PTFE) filters (5.0 µm, Whatman) using a high-volume sampler (BGI 900 BGI Inc., Waltham, MA, USA).

Particles were extracted from filters as previously described (Voss et al. 2022). Briefly, extraction was performed using pure methanol, and the particle-methanol dispersion was further sonicated and aliquoted into clean glass vials. The methanol was evaporated from the solution using a vacuum centrifuge (SpeedVac HT-4X Evaporator). Each vial contained on average 1.8 mg of eucalyptus charcoal, 3.7 mg of eucalyptus wood or 1.9 mg of dung particles.

### PAH analysis

The measurement of 21 parent PAHs (including the 16 US EPA priority PAHs), 14 alkylated species (alkyl-PAHs), 20 nitrated and 10 oxygenated PAHs (nitro-PAHs and oxy-PAHs), and 6 dibenzothiophenes (DBTs) particle-bound PAHs were performed on the collected and dried particles by gas chromatography–mass spectrometry (GC–MS), as previously described in Gren et al. (2020).

### Particle dispersion for cell exposure (sonication)

Eucalyptus charcoal and wood particles were dispersed at 1 mg/mL in endotoxin free water and sonicated for 20 and 180 min, respectively. Dung particles were first sonicated for 60 min with 200 µL of EtOH (100%) and further sonicated for 120 min after dilution to 1 mg/mL in endotoxin free water. Standard reference material (SRM) diesel exhaust PM (DEP; NIST 2975) was dispersed in endotoxin free water at 1 mg/mL and sonicated for 20 min. All particles were sonicated on ice with an ultrasonic cleaner (Branson 2200) at a frequency of 40 kHz. Particle suspensions were either used freshly prepared for cell exposure or maximum up to one week after initial preparation by re-sonication of the suspensions for 10 min.

### DLS and zeta potential

The particle size in the sonicated solution was characterized by dynamic light scattering (DLS) (Zetasizer Nano-ZS, Malvern Instruments, Worcestershire, UK). Particles were diluted at 100 µg/mL in water and cell culture medium BEGM or RPMI (in presence or absence of serum). The dispersions were measured at 25 °C directly after preparation and the average size, polydispersity index (PdI) and zeta potential were measured. Results are expressed as percentage intensity and represent the mean value of three measurements.

### Scanning electron microscopy (SEM)

Particles were dispersed in bronchial epithelial cell growth medium (BEGM, Lonza, #CC-3170). The samples were then filtered onto 0.1 µm Supor PES filters (Pall Corp.) and washed with MilliQ water before air dried. The filters were further mounted on alumina specimen pins using carbon adhesive tabs and sputter coated with a thin layer of platinum using a Q150T ES (Quorum). SEM images were acquired using an Ultra 55 field emission scanning electron microscope (Zeiss) at 3 kV using the SE2 detector.

### Acellular ROS production

To assess the intrinsic oxidative potential of the solid fuel combustion particles, the acellular particle-generated reactive oxygen species (ROS) was measured using the 2′,7′-dichlorodihydrofluorescein diacetate (DCFH-DA, Sigma-Aldrich, #287810) assay as previously described (Cediel-Ulloa et al. 2021). Briefly, particle dispersions (5–150 μg/mL) were added to a 96-well plate together with 25 μM DCF. Fluorescence was measured from the top at 485 nm excitation and 535 nm emission with a plate reader (Tecan F200) every 5 min for 30 min. The results are presented as times increase in ROS production of the mean slope per minute, normalized against the negative control (mean ± SD of two–three independent experiments).

### Endotoxin content: LAL assay

Prior to be used for cell exposure, particles were tested for endotoxin contamination using the LAL assay (Limulus Amebocyte Lysate) (Smulders et al. 2012). The endotoxin concentration in the solid fuel combustion particles was determined with the Thermo Scientific Pierce LAL Chromogenic Endotoxin Quantitation Kit (Thermo Scientific, #12117850) performed according to the manufacturer’s protocol. The levels of endotoxin measured in the particle dispersions were below 0.05 EU/mL and no interference of the particles with the LAL assay was observed.

### Cell culture and exposures

The immortalized human bronchial epithelial cell line (BEAS-2B, European Collection of Cell Cultures) was cultured in BEGM supplemented with recombinant epidermal growth factor, hydrocortisone, insulin, bovine pituitary extract, GA-1000 (Gentamicin Sulfate and Amphotericin-B), retinoic acid, transferrin, triiodothyronine, epinephrine according to manufacturers’ instructions. Cells were seeded in flasks or plates pre-coated with a mixture of 0.01 mg/mL fibronectin (Sigma-Aldrich, #F0895), 0.03 mg/mL bovine collagen type I (Sigma-Aldrich, #804622), 0.01 mg/mL bovine serum albumin (Sigma-Aldrich, #A7979) in BEGM additive free medium sub-cultured at 80% confluency. For each experiment, BEAS-2B cells were seeded one day prior particle exposure, at density of 30,000 cells/cm^2^ for 4 h, 15,000 cells/cm^2^ for 24 h and 7500 cells/cm^2^ for 48 h exposure in suitable cell culture plates.

THP-1 monocytes (ATCC) were cultured in RPMI-1640 medium (Gibco #A1049101) supplemented with 10% fetal bovine serum (FBS) and 1% Penicillin–Streptomycin (Gibco #15140122). THP-1 cells were cultured in standing cell culture T75 cm^2^ flasks and split every 3–4 days. Prior to exposure, THP-1 cells were seeded at a density of 1 × 10^5^ cells/cm^2^ in suitable cell culture plates and differentiated into macrophages by incubation with 25 ng/mL phorbol 12-myristate 13-acetate (Sigma #P8139) for 24 h.

All cells were incubated in a humidified atmosphere at 37 °C, 5% CO_2_ and regularly tested for mycoplasma contamination (MycoAlert Mycoplasma Detection Kit, Lonza, #LT07-218).

Cells were exposed to particle dispersions 24 h after seeding, at concentrations of 1 to 150 µg/mL, corresponding to 0.3 to 45.5 μg/cm^2^, in the corresponding cell medium for each cell-line. THP-1 derived macrophages were exposed to the particles in either serum-enriched (10% FBS) or serum-free medium. THP-1 cells are usually cultured in serum-enriched medium, but serum-free exposure was also included since the presence or absence of serum can affect the particle behavior (agglomeration) in suspension and by that alter the deposition and cell dose. The final exposure volumes used in 6-, 24- and 96-well plates were 2.9 mL, 0.6 mL, and 0.1 mL, respectively, in order to maintain the same μg/cm^2^ concentration between exposures. Concentration selection for cytokine release, cellular ROS production and DNA damage were done with the intention to use concentration that were primarily non-cytotoxic with the highest concentration eliciting a maximum of 20–30% cytotoxicity as per Alamar blue assay results on metabolic activity.

### Cellular morphology and particle uptake (TEM)

Transmission electron microscopy (TEM) imaging was performed as previously described (Gliga et al. 2014). In brief, BEAS-2B cells were exposed to 5 µg/mL for 24 h. Cells were then washed, harvested, separated by centrifugation, and fixed in freshly prepared 0.1 M glutaraldehyde solution. The pellets were then post fixed in 2% osmium tetroxide. Ultrathin sections (approximately 60–80 nm) were cut and contrasted with uranyl acetate followed by lead citrate. The sections were examined using a FEI Tecnai 12 Spirit Bio TWIN transmission electron microscope at 100 kV.

### Metabolic activity evaluation (Alamar blue assay)

BEAS-2B cells or THP-1 derived macrophages were seeded in transparent 96-well plates and exposed to the particle dispersions at concentrations ranging from 1 to 150 μg/mL for 24 or 48 h. After exposure, exposure medium was replaced with cell medium with 10% AlamarBlue reagent (AlamarBlue Cell Viability Reagent, ThermoFisher Scientific, #A50100) and incubated for 2 h at 37 °C. The fluorescence was measured at 560 nm excitation and 590 nm emission wavelengths using a plate reader (Tecan Infinite F200). Cells were exposed to 5% and 10% of DMSO as positive controls. Cells were exposed to vehicle control (water and ethanol at the highest concentration present in the particle suspensions for exposure, i.e., 10% water, 1% ethanol in BEGM media). No difference in metabolic activity was observed between unexposed cells and cells exposed to the vehicle control (data not shown). Results were expressed as percentage metabolic activity with the negative control set to 100% metabolic activity. The experiments were performed in triplicate wells for each time point and concentration. Results are presented as mean values ± SD of three independent experiments.

### Cell viability (cellular membrane damage—LDH release)

The Cytotox 96 Non-Radioactive Cytotoxicity Assay Kit (Promega) was used in a 96-well plate format. BEAS-2B cells were exposed to particle dispersions at concentrations ranging from 1 to 150 μg/mL in 100 μL for 24 h and 48 h. After exposure, the supernatant was transferred to a 96-well v-bottom plate. Cells were washed twice with PBS and then lysed with 100 μL Triton 1% for 30 min at 37 °C. Supernatants and cell lysates were separated by centrifugation for 5 min at 3000 g and 50 µL of supernatant was collected in a new 96-well plate and incubate with 50 μL of reconstituted substrate for 30 min. Reactions were terminated using 50 μL stop solution and absorbance was measured at 495 nm using a plate reader (Tecan Infinite F200). The absorbance of the supernatant corresponds to LDH release, whereas the sum of the absorbance of the supernatant and cell lysate corresponds to the maximum LDH release. The cell viability was calculated by dividing the LDH release to the maximum LDH release for each well. The control was set to 100% viability and the results were expressed as percentage cell viability. The experiments were performed in triplicate wells for each time point and concentration. Particle interference with the LDH assay was tested by incubating particles with the cell culture media or with cell lysates and LDH assay was further performed as described above. The acellular interference was performed by incubating different concentrations of particles with reconstituted LDH substrate. No interference with the assay was detected. Results are presented as mean values ± SD of three independent experiments.

### Cellular ROS production (DCFH-DA assay)

The 2′,7′-dichlorodihydrofluorescein diacetate (DCFH-DA) assay was used to measure intracellular ROS production as previously described (Gliga et al. 2014). BEAS-2B cells were seeded in black 96-well plates with transparent bottom (Corning Incorporated) in triplicates for each exposure. 24 h after seeding, cells were incubated with 20 μM DCFH-DA (Sigma-Aldrich, #D6883) for 45 min. Cells were afterwards washed once with Hanks’ Balanced Salt Solution (HBSS, Sigma-Aldrich, #H8264) and then exposed to particles at non- or sub-toxic concentrations (1–25 µg/mL) for 4 h. For measurement of the ROS production, cells were washed with Hanks’ Balanced Salt Solution, and fluorescence was directly recorded (excitation 485 nm, emission 530 nm). Cells were exposed to 100 µM Tert-butyl hydroperoxide (Sigma-Aldrich, #458139) as positive control. Fluorescence was recorded every 5 min for 30 min (excitation 485 nm, emission 535 nm) using a plate reader (Tecan Infinite F200, Software: Magellan 7.2, Austria) at 37 °C and ROS increase was calculated as mean slope per min and normalized to the unexposed control. Results are presented as mean values ± SD of three independent experiments.

### Genotoxicity (alkaline comet assay)

Genotoxicity was evaluated by using the mini-gel version of the alkaline comet assay as previously described (Di Bucchianico et al. 2017). Cells were seeded in 24-well plates and exposed to particle dispersions. In BEAS-2B, the concentrations ranged from 5 to 25 μg/mL (dung, eucalyptus wood) or 5 to 100 μg/mL (eucalyptus charcoal, DEP) for 4 h and 24 h. THP-1 derived macrophages were exposed for 24 h at concentrations from 10 to 100 μg/mL in serum-free conditions and 100 μg/mL in the presence of serum. DNA was stained by immersing slides in 1:10 000 SYBR-green in TAE buffer for 15 min. Each sample was run in duplicate gels and at least 50 nucleoids were scored per gel using a fluorescence microscope (Leica DMLB) with Comet assay VI software (Perspective Instruments). Results are presented as % DNA in tail based on mean values ± SD of two–three independent experiments.

### Pro-inflammatory response analysis (cytokine release measurement)

Secretion of the cytokines and interleukins IL-6, IL-8 (CXCL8), IL-12p70, IL-4, IL-1β and TNF-α was quantified by multiplex electrochemiluminescence immunoassay in supernatants of BEAS-2B following short term (24 h) exposure to 1, 10, 25 (all) and 100 μg/mL (eucalyptus charcoal) biomass combustion particles. Secretion of cytokines and interleukins IL-6, IL-8, IL-1β and TNF- α was similarly quantified in supernatants of THP-1 derived macrophages following 24 h exposure to 25, 50 and 100 μg/mL biomass combustion particles in serum-free conditions and 100 μg/mL biomass combustion particles in serum-enriched medium. Exposure to lipopolysaccharide (LPS) was included as a positive control for BEAS-2B (1 µg/mL, 8 h) and THP-1 derived macrophages (100 ng/mL, 24 h).

Each plate was analyzed on the Meso QuickplexSQ120 (MesoScale Discovery) according to manufacturer’s instructions for 96-well plates to obtain absolute protein levels (pg/mL). IL-12p70, IL-4, IL-1β and TNF-α concentrations were below the limit of quantification in the negative controls for BEAS-2B cells and therefore excluded from the analysis. Results are expressed as fold change compared to negative control and presented as mean values ± standard deviation (SD) of three independent experiments.

### Statistical analysis and data processing

One-way ANOVA followed by Dunnett’s post hoc test was used for comparison between the treatments and the negative control. For results of the comet assay and cytokine secretion, complementary two-tailed unpaired t-test was performed between treatments and negative control or between treatments. If only one exposure concentration was used, two-tailed unpaired t-test was performed between the treatments and the negative control or between treatments. Linear regression was performed for each exposure group to determine any linear trends for acellular ROS, DNA damage and cytokine secretion as determined based on applied concentrations.

The half maximal effective concentration (EC50) for each particle type was estimated for metabolic activity and LDH release data by performing a dose–response analysis as described in Dubey et al*.* (2015). Briefly, particle concentrations were log-transformed and EC50 calculated with nonlinear regression analysis. All analyses and graphs were performed using GraphPad Prism 9.0.0 for Windows, GraphPad Software, San Diego, California USA. *P*-values < 0.05 were considered statistically significant.

A qualitative relative comparison was done of the results of particle characterization (PAH content and acellular ROS) and toxicity endpoints by categorizing the outcome into no/low/medium/high content/response. The categorization was done by weighing in several factors including significant difference from control at one or more concentrations, linear trends, fold change compared with unexposed control and number of significant cytokines (only for secretion of inflammatory cytokines).

## Results

### Physico-chemical properties of the combustion particles in suspension

**Fig. 1:**
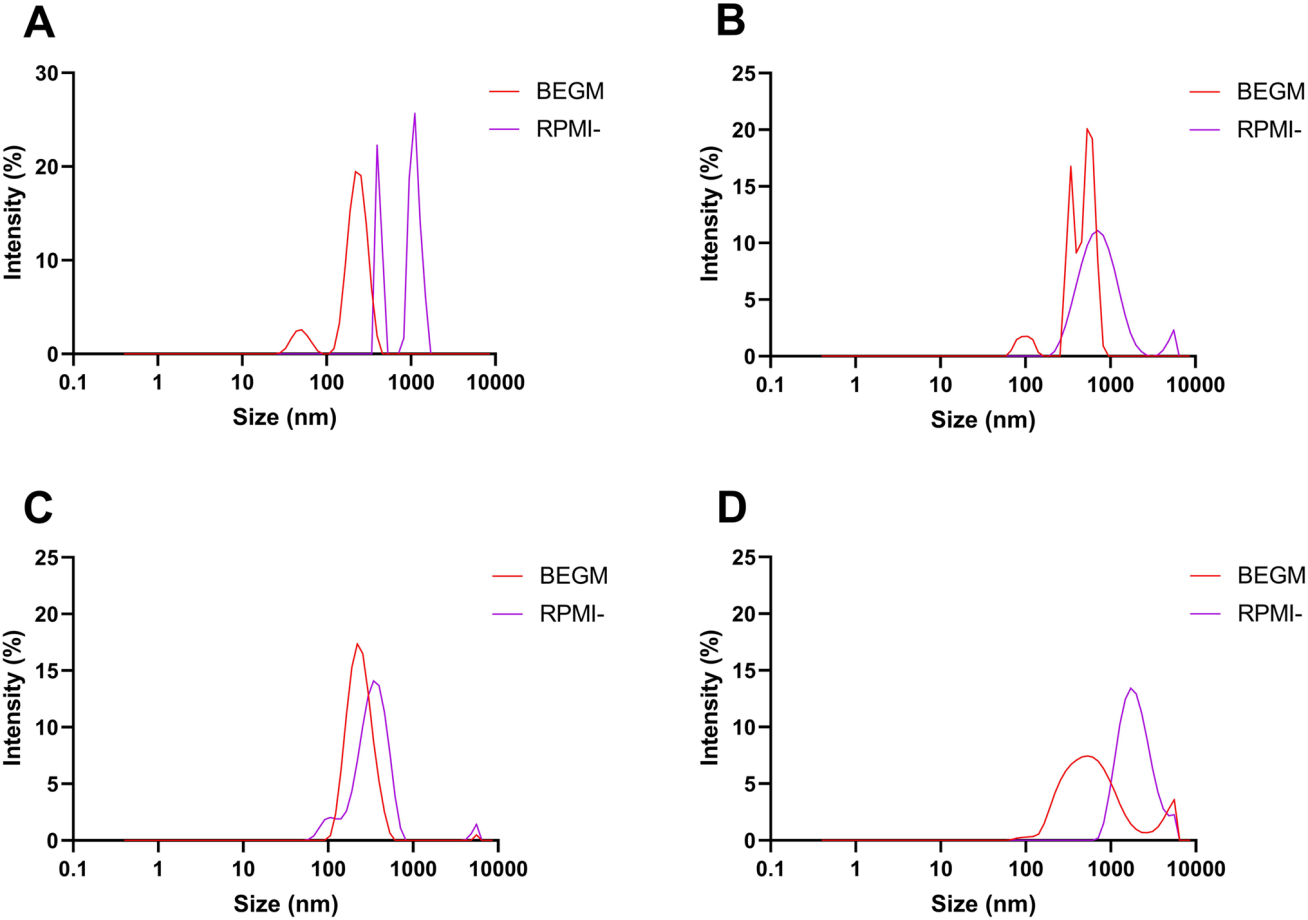
Hydrodynamic size distribution of particles generated from combustion of solid fuel (**A** dung, **B** eucalyptus wood, **C** eucalyptus charcoal) or diesel exhaust particles (D) dispersed (100 µg/mL) in BEGM or serum-free RPMI (RPMI-). Data is evaluated by dynamic light scattering and expressed as intensity (%)

Size of particle agglomerates was evaluated by DLS in water, BEGM (BEAS-2B cell medium) and RPMI with or without serum (THP-1 cell medium). Combustion particles from dung had a polydispersity index close to 1 in both water and RPMI without serum, which indicates a very heterogenous dispersion (Table 1). Similarly, combustion particles from wood displayed a polydispersity index of 0.8 in BEGM. The remaining particle suspensions had a more homogenous dispersion with a polydispersity index of 0.5 and below. Overall, particles agglomerated to a higher extent in the serum-free RPMI medium compared with the serum supplemented RPMI. Histograms of the size distributions are shown in Fig. 1 and supplementary Figure S1 indicates a multi-modal size distribution pattern. Surface charge in water was positive for combustion particles from dung but negative for those derived from wood and charcoal (Table 1).

**Table 1:** Particle characterization of particles generated from combustion of solid fuels or diesel exhaust particles (DEP, NIST 2975) by dynamic light scattering (DLS)

Particles	Water				BEGM			RPMI + FBS			RPMI-FBS		
	Z-average	PdI	Main peak	Z-potential	Z-average	PdI	Main peak	Z-average	PdI	Main peak	Z-average	PdI	Main peak
	(nm)		(nm)		(nm)		(nm)	(nm)		(nm)	(nm)		(nm)
Dung	1367	0.9	477	3.1	290	0.3	238	7151	0.5	426	882	0.9	892
Wood	792	0.3	808	− 16.6	1089	0.8	499	719	0.3	507	378	0.4	777
Charcoal	242	0.4	248	− 30.1	237	0.4	245	399	0.4	297	144	0.4	349
DEP	172	0.3	332	N/A	412	0.4	711	1760	0.3	220	188	0.1	2025

Particles were dispersed at 100 µg/mL in water, BEGM cell culture medium or RPMI-1640 cell culture medium with or without serum (10% fetal bovine serum FBS)

*PdI* polydispersity index

**Fig. 2:**
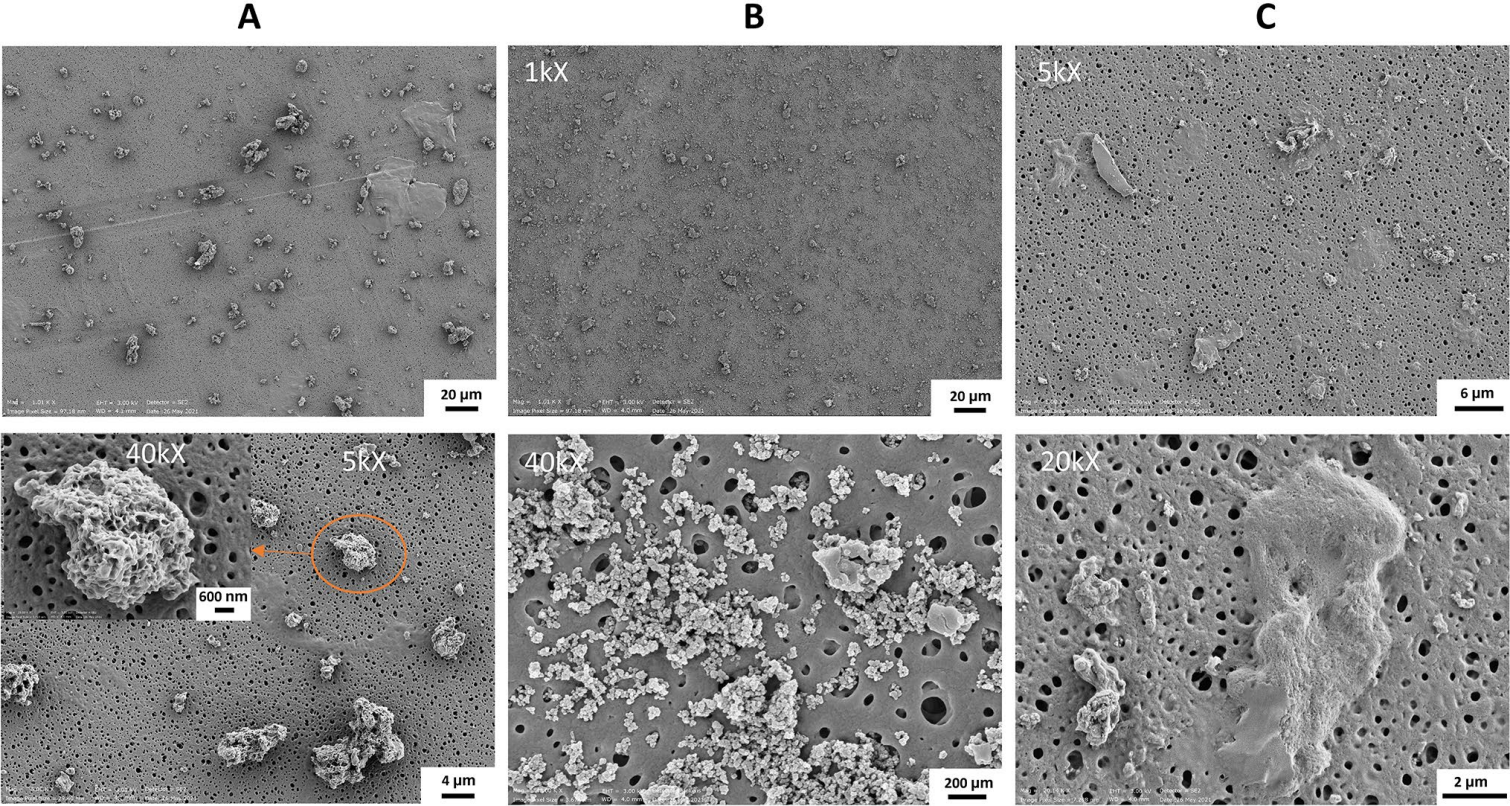
Scanning electron microscopy images of particles generated from combustion of solid fuel (**A** dung, **B** eucalyptus wood, **C** eucalyptus charcoal) dispersed in cell media (BEGM)

The particle dispersion in BEGM were additionally visualized by SEM. The cow dung combustion particles presented a non-spherical shape, a porous surface and sizes ranging from a few hundred nanometers to 10 µm (Fig. 2A, upper and lower insert). The eucalyptus wood combustion particles were spherical, had a smooth surface and formed large agglomerates while seemingly preserving the individual particle identity (Fig. 2B, upper and lower insert). The size of the eucalyptus wood particles ranged from below 100 nm up to approximately 10 µm (Fig. 2B, upper and lower insert). The eucalyptus charcoal particles had a non-spherical shape, a porous surface, and a wide size distribution (100 nm–5 µm) (Fig. 2C, upper and lower insert).

**Fig. 3:**
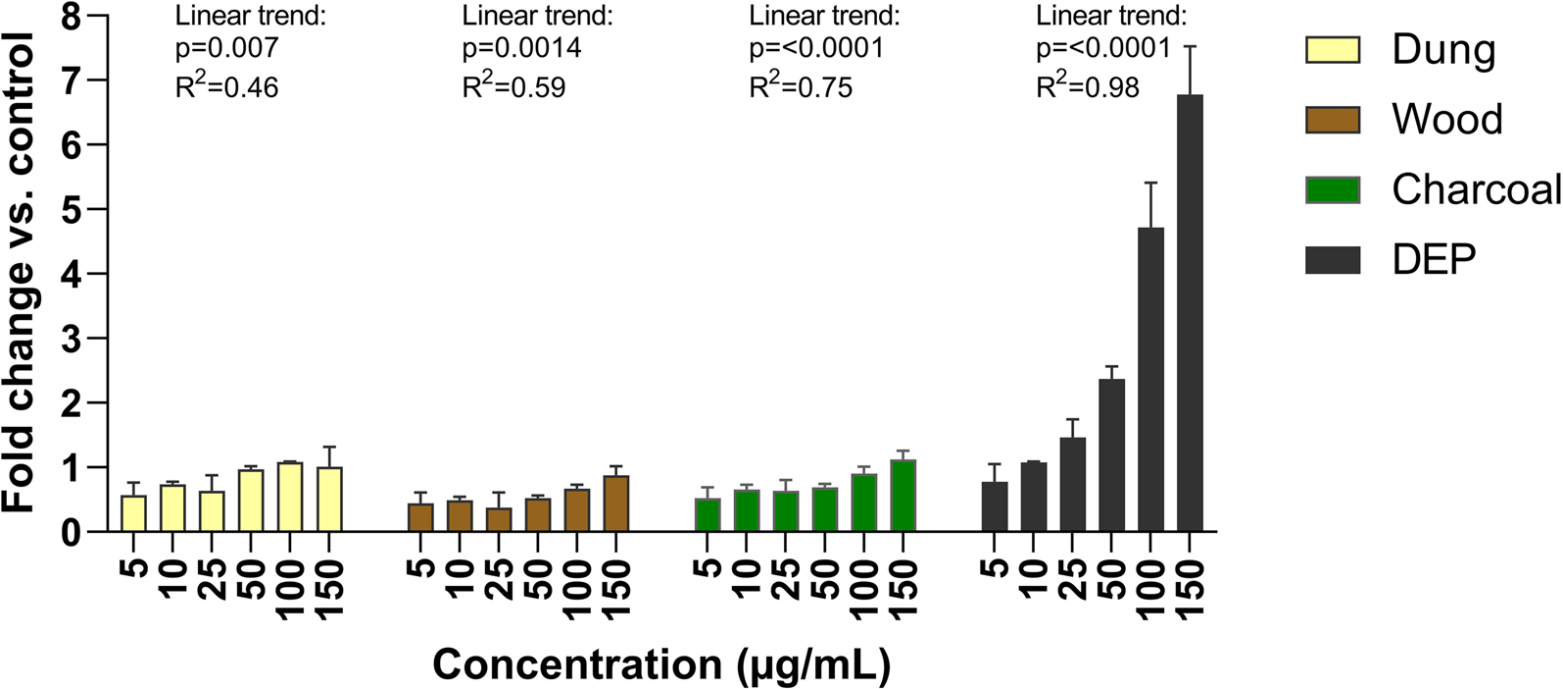
Generation of reactive oxygen species (ROS) in BEAS-2B medium. Particles generated from combustion of solid fuel (dung, eucalyptus wood, eucalyptus charcoal) or diesel exhaust particles were dispersed and diluted in cell medium (BEGM). Production of ROS was evaluated in acellular condition using the modified DCFH-DA assay as described in methods. Results are expressed as fold change versus untreated control. Results are presented as mean ± S.D. (*n* = 2–3). Linear trends were determined by simple linear regression and displayed above each exposure group if significant (*P* < 0.05)

The generation of acellular ROS was determined for particle suspensions in BEGM cell medium. A significant positive linear trend was observed for all combustion particles and DEP (Fig. 3), where reference DEP particles showed a considerably higher response compared with the combustion particles.

Size distribution and particle composition of the airborne particles, as well as emission factors and exposure levels were previously reported by Eriksson et al. (2022).

### Particulate PAH content

The PAH composition of the collected particles after methanol evaporation are presented in Table 2. Table S1 contains information on individual PAHs. All samples contained parent, oxy- and nitro-PAHs. The total particulate PAH mass fraction was highest for particles derived from wood (3219 ng/mg), followed by dung (618 ng/mg), charcoal (135 ng/mg) and DEP (118 ng/mg). The wood combustion particles also had the highest mass fraction of native PAHs (2831 ng/mg) and oxy-PAHs (239 ng/mg). We found comparable mass fractions of alkyl-PAHs between dung (99 ng/mg) and wood (105 ng/mg) combustion particles. Particles derived from dung had the highest mass of nitro-PAHs (89 ng/mg), while comparable levels of nitro-PAHs were detected in wood (44 ng/mg) and DEP (48 ng/mg) combustion particles. DBTs, both native and alkylated, were detected in particles at very low concentrations (less than 1% of the total PAH mass fraction), with the highest mass found in particles emitted from dung combustion (4.6 ng/mg).

**Table 2:** Composition of polycyclic aromatic hydrocarbons (PAHs) of the extracted PM samples (ng/mg particles) generated from combustion of solid fuels or diesel exhaust particles (DEP, NIST 2975)

	Dung		Wood		Charcoal		DEP	
	ng/mg	%	ng/mg	%	ng/mg	%	ng/mg	%
PAHs	351.6	56.9	2831.0	88.0	114.7	84.7	41.3	35.1
Alkyl-PAHs	99.0	16.0	104.7	3.3	7.4	5.5	6.8	5.8
DBTs	4.6	0.7	0.7	0.0	0.3	0.2	1.0	0.9
Nitro-PAHs	88.7	14.4	43.7	1.4	5.7	4.2	48.0	40.9
Oxy-PAHs	73.9	12.0	238.6	7.4	7.3	5.4	20.4	17.3
Total	617.7	100.0	3218.6	100.0	135.5	100.0	117.6	100.0

The PAHs are given in groups of native PAHs and PAH derivatives: alkylated species (alkyl-PAHs), dibenzothiophenes (DBTs), nitrated and oxygenated PAHs (nitro-PAHs and oxy-PAHs). A full list of measured individual compounds can be found in Table S1

Figure S2 shows the content of PAHs segregated into low (2–3 rings), middle (4 rings and retene, which is 3 rings but with side chains) and high (5–6 rings) molecular weight isomers (see Table S1 for the grouping of PAHs). This distinguishes the wood combustion particles as the sample with the largest fraction of high molecular weight PAH, followed by those derived from cow dung.

### Combustion particles derived from dung and eucalyptus wood were more cytotoxic compared with those from eucalyptus charcoal

**Fig. 4:**
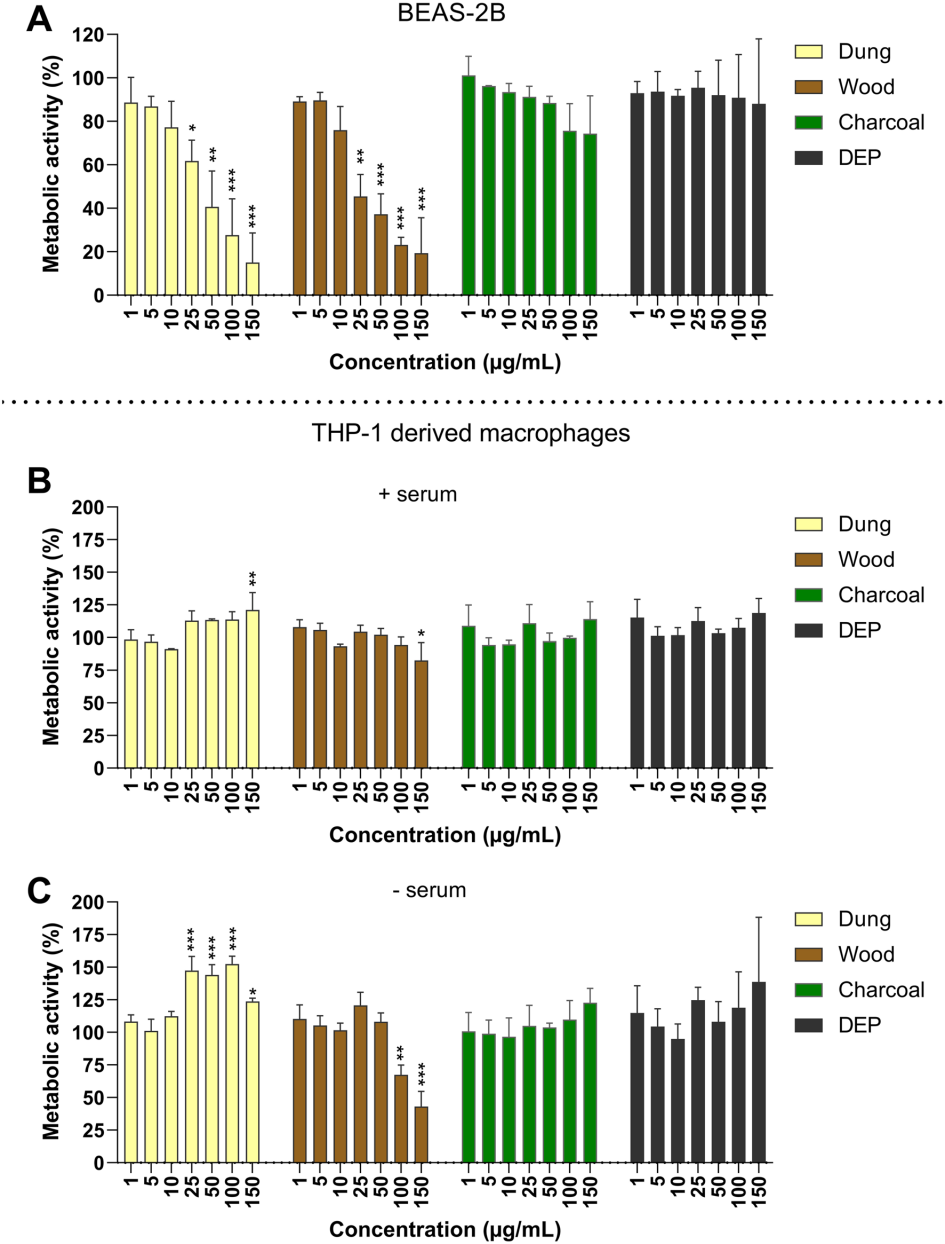
Cytotoxicity of combustion particles in bronchial epithelial cells and monocyte derived macrophages. BEAS-2B (**A**) or differentiated THP-1 cells (**B, C**) were exposed to particles generated from combustion of solid fuel (dung, eucalyptus wood, eucalyptus charcoal) or diesel exhaust particles for 24 h. THP-1 were exposed in serum-enriched (**B**) or serum-free (**C**) cell medium. Cell viability was assessed using Alamar Blue assay and results are expressed as % metabolic activity as compared to the untreated control. Results are presented as mean ± S.D. (*n* = 3). Statistically significant differences, as compared to the control are labelled with asterisks (* for *P*-value < 0.05, ** for *P*-value < 0.01, *** for *P*-value < 0.001)

Cytotoxicity of the biomass combustion particles in BEAS-2B bronchial cells and THP-1 derived macrophages was expressed as metabolic activity (Fig. 4, S3A) and cell viability (Figure S3B, C) after exposure for 24 and 48 h. Exposure to dung and wood combustion particles reduced the metabolic activity of BEAS-2B in a concentration- and time-dependent manner (Fig. 4A, Figure S3A). A significant reduction in metabolic activity was observed from concentrations of 25 µg/mL following 24 h exposure for both dung and wood combustion particles, and from 5 and 10 µg/mL after 48 h, respectively. Eucalyptus charcoal combustion particles did not affect the metabolic activity of BEAS-2B after 24 h, but a significant reduction was observed after 48 h at high concentrations (100–150 µg/mL) (Figure S3A).

Cell viability, measured by the LDH assay, of BEAS-2B was significantly reduced after 24 h exposure to dung and eucalyptus wood combustion particles at exposure concentrations of 50 µg/mL and higher (Figure S3B). After 48 h, a reduction in cell viability was observed from concentrations of 25 µg/mL or 50 µg/mL for the wood and dung combustion particles, respectively (Figure S3C). There was no reduction in cell viability after exposure to particles derived from eucalyptus charcoal under the test conditions (Figure S3B, C). In addition, the reference DEP particles induced no alteration of the metabolic activity or cytotoxicity (Fig. 4A, Figure S3A-C).

No decrease in metabolic activity was observed in THP-1 derived macrophages after exposure to combustion particles or DEP in serum-supplemented cell medium (24 h, Fig. 4B). At serum-free conditions, combustion particles from wood elicited a significant decrease in metabolic activity at concentrations > 100 µg/mL in THP-1 derived macrophages but no decrease were observed for the remaining combustion particles or DEP (24 h, Fig. 4C). Instead, exposure to combustion particles derived from dung resulted in an increase in metabolic activity at concentrations of 25 µg/mL and above, as compared with negative control.

EC50 values for metabolic activity and cytotoxicity are presented in Table S3. Based on metabolic activity, the wood combustion particles demonstrated the lowest cytotoxic EC50 values in BEAS-2B of 11 and 28 µg/mL at 48 and 24 h, respectively. The corresponding EC50 values for the cytotoxicity of dung combustion particles were 35 and 16 µg/mL. Based on the LDH assay in BEAS-2B, combustion particles from dung and wood had comparable EC50 values at 48 h of 82 and 88 µg/mL, respectively. Overall, EC values were lower for metabolic activity compared with the cell viability for the biomass combustion particles tested in BEAS-2B (Table S3). The EC50 value for wood combustion particles in THP-1 derived macrophages was 133 µg/mL, based on metabolic activity, while the remaining exposures had a EC50 value > 150 µg/mL in both serum-enriched and serum-free conditions.

We also used TEM to evaluate cell morphology at low or non-cytotoxic concentrations (5 or 25 µg/mL) in BEAS-2B, see Figure S4. No morphological difference could be observed after treatment of BEAS-2B with the biomass combustion particles. Some particles could be distinguished outside or inside the cells following exposure to wood combustion particles and DEP, as observed in membrane-bound structures inside the cytoplasm (Figure S4, see arrows). No particles could be distinguished inside the cells after exposure to combustion particles from dung and charcoal, but that should be interpreted cautiously since the biomass combustion particles contain organic material which may make them difficult to be distinguished in organic samples.

### Biomass combustion particles induce DNA damage in human bronchial cells and monocyte derived macrophages

Induction of DNA damage in BEAS-2B bronchial cells (4 and 24 h) and THP-1 derived macrophages (24 h) following exposure to biomass particles was evaluated by the alkaline comet assay. After 4 h exposure in BEAS-2B, biomass particles from wood and charcoal at the concentration of 25 µg/mL and the DEP at the highest concentration (100 µg/mL) induced a significant increase of DNA damage, as compared with negative control (Figure S5). Significant linear trends were observed for particles derived from wood, charcoal, and DEP, but not for charcoal combustion particles. In addition, we also investigated the generation of cellular ROS after 4 h exposure in BEAS-2B. None one of the biomass combustion particles increased the cellular production of ROS at concentrations up to 25 µg/mL (Figure S6).

**Fig. 5:**
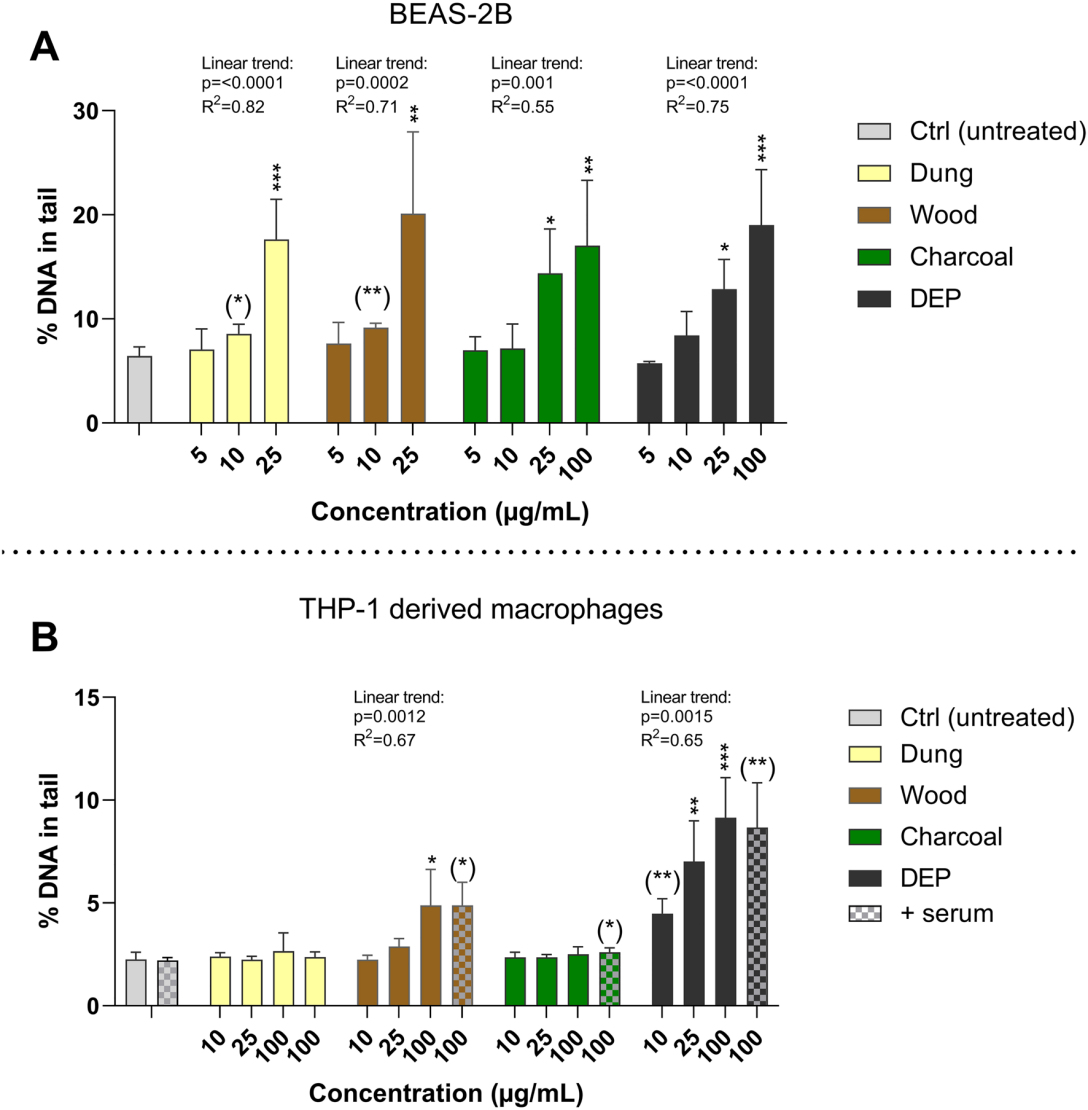
Induction of DNA damage in human bronchial cells or monocyte derived macrophages exposed to combustion particles. Induction of DNA strand breaks following 24 h exposure to particles generated from combustion of solid fuel (dung, eucalyptus wood, eucalyptus charcoal) or diesel exhaust particles was quantified by the comet assay under alkaline conditions in BEAS-2B (**A**) or THP-1 derived macrophages in serum-free (no pattern) or supplemented (patterned) medium (**B**). DNA damage was quantified as percent of DNA in the comet tail. Results are presented as mean values ± S.D. (*n* = 3, *n* = 2 for charcoal 100 µg/mL in THP-1 without serum). Statistically significant differences, as compared to the control by one-way ANOVA followed by Dunnett’s are labelled with asterisks (* for *P*-value < 0.05, ** for *P*-value < 0.01, *** for *P*-value < 0.001). Statistically significant differences between treatments and control as determined by two-tailed unpaired t-test are labelled with (*). Linear trends were determined in BEAS-2B or THP-1 in serum-free conditions by simple linear regression and displayed above each exposure group if significant (*P* < 0.05)

Following 24 h exposure in BEAS-2B, all particles tested induced DNA damage in a concentration-responsive manner with significant linear trends (Fig. 5A). A significant increase in DNA damage was observed at concentrations starting from 10 µg/mL for biomass combustion particles from dung and wood, and from 25 µg/mL for those generated by eucalyptus charcoal combustion and the reference DEP. At the concentration of 25 µg/mL, biomass particles from dung and eucalyptus wood resulted in a threefold increase whilst the exposure to biomass particles from eucalyptus charcoal and DEP induced a twofold increase, as compared with negative control. Generation of ROS was investigated with the DCFH-DA assay after short-term (4 h) exposure in BEAS-2B cells (Figure S6).

Following 24 h exposure in THP-1 derived macrophages in serum-free conditions (Fig. 5B), particles derived from wood induced DNA damage at the highest concentration tested (100 µg/mL) and displayed a significant linear trend. Exposure to DEP resulted in a significant increase for all concentration tested (10–100 µg/mL) in a concentration-dependent manner with a significant linear trend. No effects were seen in response to dung or charcoal combustion particles in serum-free conditions. In serum-supplemented cell medium (Fig. 5B), particles derived from wood and charcoal as well as DEP induced a significant increase in DNA damage in THP-1 derived macrophages at the single concentration tested of 100 µg/mL. Dung-derived particles did not induce genotoxic effects in serum-supplemented conditions at the concentration tested (Fig. 5B). No significant differences were seen in the genotoxic response between THP-1 derived macrophages being exposed to combustion particles (100 µg/mL) in serum-free or serum-enriched cell medium.

### Biomass combustion particles induce secretion of pro-inflammatory cytokines in monocyte derived macrophages but not in bronchial epithelial cells

**Fig. 6:**
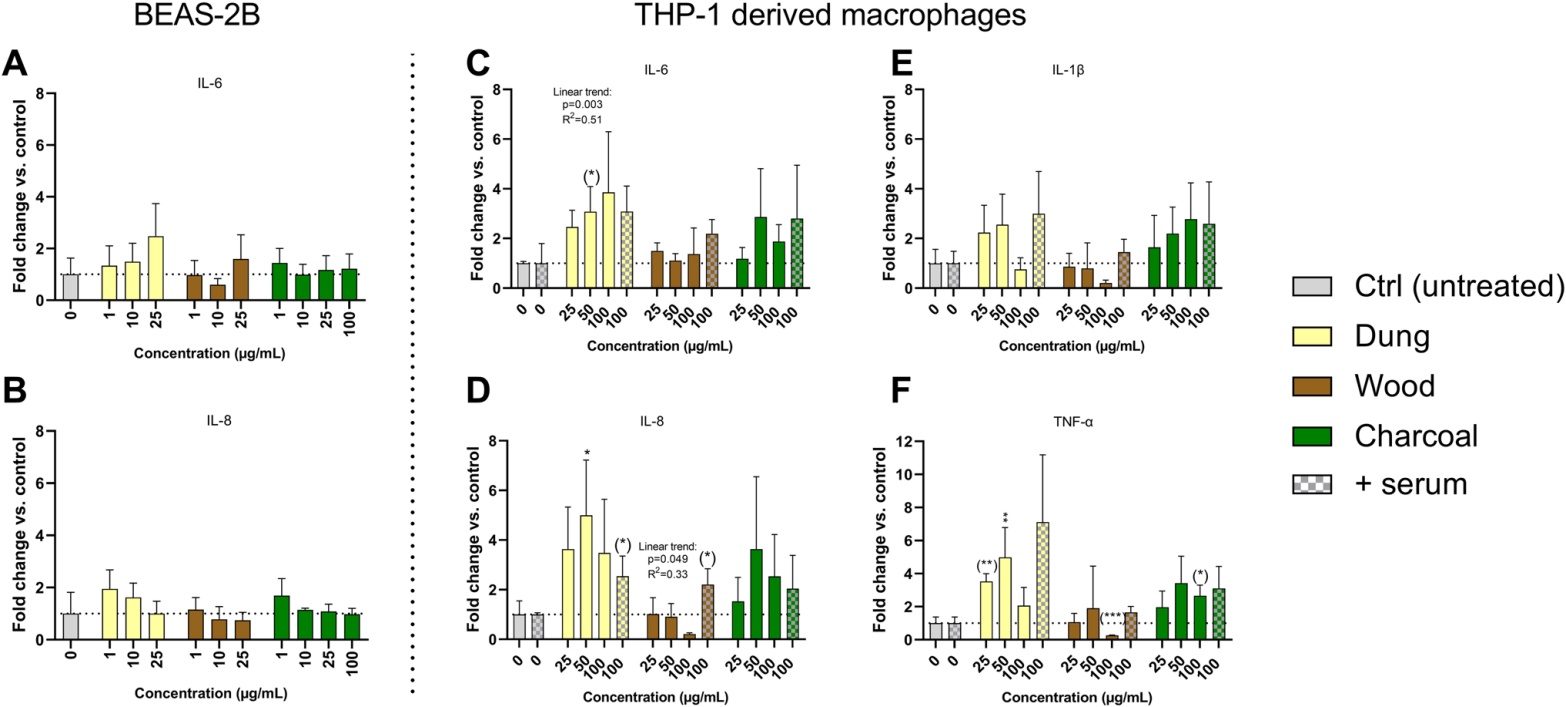
Secretion of cytokines in bronchial cells or monocyte derived macrophages exposed to combustion particles. Secretion of cytokines following 24 h exposure to particles generated from combustion of solid fuel (dung, eucalyptus wood, eucalyptus charcoal) or diesel exhaust particles in BEAS-2B (**A, B**) or differentiated THP-1 in serum free (no pattern) or supplemented (patterned) cell medium (**D**–**F**) was quantified by multiplex electrochemiluminescence immunoassay. Exposure to LPS (1 µg/mL, 8 h for BEAS-2B, 100 nM 24 h for THP-1) induced > 40-fold increase for all cytokines included. Results are presented as mean values of fold change compared to negative control ± S.D. (*n* = 3). Statistically significant differences, as compared to the control by one-way ANOVA followed by Dunnets are labelled with asterisks (* for *P*-value < 0.05, ** for *P*-value < 0.01, *** for *P*-value < 0.001). Statistically significant differences between treatments and control as determined by two-tailed unpaired t-test are labelled with (*). Linear trends were determined in BEAS-2B or THP-1 in serum-free conditions by simple linear regression and displayed above each exposure group if significant (*P* < 0.05)

We evaluated the secretion of pro-inflammatory cytokines in BEAS-2B bronchial cells and THP-1 derived macrophages following 24 h exposure to non- or subtoxic concentrations of combustion particles (Fig. 6). None of the tested particles altered the secretion of IL-6 and IL-8 in BEAS-2B at concentrations up to 25 (dung, wood) or 100 (charcoal) µg/mL, as compared with negative controls (Fig. 6A, B).

In THP-1 derived macrophages, combustion particles from dung significantly increased the secretion of IL-6 (Fig. 6C), IL-8 (Fig. 6D) and TNF-α (Fig. 6F) under serum-free conditions, with a significant positive linear trend for IL-6. Combustion particles from charcoal increased TNF-α at the highest concentration tested (100 µg/mL) (Fig. 6F). Particles derived from wood did not induce secretion of any of the cytokines tested in THP-1 cells in serum-free medium (Fig. 6C–F). Instead, exposure to wood-derived combustion particles significantly decreased the secretion of TNF- α (100 µg/mL) and resulted in a negative linear trend for IL-8. Combustion particles did not induce IL-1β at serum-free exposure.

In serum-enriched cell medium, particles derived from dung and wood elicited a significant increase in secretion of IL-8 at the single concentration tested of 100 µg/mL (Fig. 6D). No effects on IL-6, IL-1β or TNF-α were observed under serum-enriched conditions for any of the combustion particles (Fig. 6C, E, F). A significant difference was seen in the cytokine response between cells exposed to wood combustion particles (100 µg/mL) in serum-free or serum-enriched medium for IL-8, IL-1β and TNF-α (*P* < 0.05). No difference was seen in the response between exposure with or without serum for dung or charcoal-generated combustion particles.

## Discussion

In this study, we explored the toxicity of PM_2.5_ emitted from combustion of the solid biomass fuels eucalyptus wood, eucalyptus charcoal and cow dung, which are frequently used in households in sub-Saharan Africa. Our results demonstrate that PM_2.5_ generated from the combustion of these traditional biomass fuels are cytotoxic, induce DNA damage and trigger pro-inflammatory cytokine secretion after short-term exposure in human bronchial epithelial cells and/or monocyte derived macrophages. The particles originating from the less energy dense solid fuels, such as wood and dung, had higher PAH content and were more potent at inducing cytotoxicity in lung epithelial cells compared with particles generated from higher energy dense fuels, such as charcoal and diesel. The dung particles originating from the least energy dense fuel, dried cow dung, were the only ones to increase secretion of pro-inflammatory cytokines in macrophage-like cells. This suggests that the typeof solid biomass fuel impacts the toxicological profile of the combustion particles. See Table 3 for a qualitative relative comparison of the particles.

**Table 3:** Summary of the results as qualitative relative comparison of endpoints for particles generated from combustion of traditional Ethiopian fuel and the reference diesel exhaust particles (DEP, NIST 2975)

Toxic endpoint/characterisation	Energy density and price of fuel			
	Cow dung	Eucalyptus wood	Eucalyptus charcoal	DEP
Total PAH content	+ +	+ + +	+	+
Acellular ROS	+	+	+	+ + +
BEAS-2B				
Cytotoxicity	+ + +	+ + +	–	–
DNA damage	+ + +	+ + +	+ + +	+ + +
Secretion of inflammatory cytokines	–	–	–	N/A
THP-1 (serum enriched)				
Cytotoxicity	–	+	–	–
DNA damage	(–)	(+ +)	(–)	(+ + +)
Secretion of inflammatory cytokines	(+)	(+)	(–)	N/A
THP-1 (serum free)				
Cytotoxicity	–	+ +	–	–
DNA damage	–	+	–	+ + +
Secretion of inflammatory cytokines	+ + +	–	+	N/A

The outcomes (characterization and toxicity) were categorized into no/low/medium/high content/response by weighing in several factors including magnitude, significant difference from control and dose response trends

Relative categories: “–” = no content/response, “+” = low content/response, “+ +” = medium content/response, “+ + +” = high content/response, brackets = only one tested concentration, N/A = not analyzed, *PAH* polycyclic aromatic hydrocarbons

The combustion of dung, wood and charcoal led to the generation of PM_2.5_ of variable chemical and physical properties, which could explain their different toxic potential. Particles generated from the combustion of dung and wood had a similar cytotoxic profile in BEAS-2B cells being considerably more cytotoxic (EC50 < 35 µg/mL, metabolic activity 24 h) compared with those derived from eucalyptus charcoal and DEP, which demonstrated no or low cytotoxic potency (EC50 > 150 µg/mL, metabolic activity 24 h). In THP-1 derived macrophages exposed at serum-free conditions, only wood combustion particles induced a cytotoxic response (EC50 133 µg/mL, metabolic activity 24 h), whereas no effects were seen for any of the particles in the presence of serum. The difference in cytotoxic potential between the combustion particles could, to some extent, be explained by the different PAH compositions. Wood particles had approximately fivefold higher total PAH content than dung, which in turn had approximately fivefold higher PAH content compared to charcoal. Of those PAHs, high molecular weight PAHs were 14-fold higher in wood particles compared to dung particles, and 33-fold higher compared to eucalyptus particles. High concentrations of PAHs in PM have previously been associated with apoptosis in vitro (Uski et al. 2014; Yang et al. 2016; Marchetti et al. 2019), which could possibly explain the high cytotoxic potential of biomass particles from dung and wood. In addition, particles derived from dung and wood had higher levels of several groups of PAH derivatives including alkyl-PAHs and oxy-PAHs. Much less is known about the toxic potential of alkyl-, nitro- and oxy-PAHs, but some have been shown to affect human health upon exposure as direct acting mutagens and carcinogens. Several PAH derivatives have been shown to exhibit even higher toxicity than their parent PAHs or benzo[a]pyrene (Durant et al. 1996; Clergé et al. 2019; McCarrick et al. 2019).

All particles tested, including the reference DEP, induced DNA damage in BEAS-2B bronchial cells. Following 4 h of exposure, wood and charcoal combustion particles elicited genotoxicity at the lowest concentration but after 24 h, all particles showed a similar genotoxicity profile, independent of their large differences in PAH content. This indicates that the total PAH content is a more important determinant for cytotoxicity, rather than genotoxicity in BEAS-2B cells in the context of this study. A higher PAH content does not automatically equate to higher DNA damage, since high levels of DNA damage per se could trigger cell death, and this DNA damage would not be captured in the comet assay. In THP-1 derived macrophages, particles derived from wood were the only combustion particles found to induce genotoxicity (serum-free conditions) which could potentially be influenced by the high PAH content. Generally, the genotoxicity of PM from HAP has not been studied extensively in vitro, especially those of relevance for sub-Saharan Africa. Our results are in agreement with previous in vitro studies reporting genotoxic effects of various types of wood combustion particles (Leonard et al. 2000; Danielsen et al. 2009; Tapanainen et al. 2012; Arif et al. 2017; Marabini et al. 2017). In a study by Kim et al. (2018), the toxicity of combustion particles from different types of wood were compared in vitro using human lung cells; the greatest lung toxicity was observed for eucalyptus, but the greatest mutagenicity for pine wood. Cooking with biomass has further been associated with DNA damage in buccal and sputum cells in non-smoking women in rural India (Mondal et al. 2010, 2011; Mukherjee et al. 2013).

The reference DEP particles induced DNA damage in both cell lines tested in this study. Danielsen et al. (2009) reported birch wood smoke PM to induce a greater response of DNA strand breaks and oxidative DNA damage in both alveolar A549 cells and THP-1 monocytes compared to the same DEP (NIST 2975) as used in our study. This is in contrast to our results, demonstrating comparable (BEAS-2B) or more elevated (THP-1) genotoxic response in response to DEP compared to wood combustion particles. Yet, it is important to consider that the wood combustion particles were generated under different setups and stoves. It has been shown that tumorigenic PAHs require metabolic activation to elicit their effect and the genotoxic sensitivity towards PAHs may therefore be largely different between cell lines due to metabolic capacity (Moorthy et al. 2015; Shah et al. 2016; Oesch et al. 2019). The inclusion of a cell model with more extensive CYP metabolism, such as the hepatic cell line HepG2, might be interesting to further study the genotoxic effects of the solid biomass combustion particles (Knasmüller et al. 1998; Garcia-Canton et al. 2013). It is also important to note that this study is performed under acute conditions, and more long-term studies on the genotoxic effects would also be relevant.

No inflammatory effects in terms of pro-inflammatory cytokine release were observed in response to combustion particles in BEAS-2B. By using the more immunocompetent THP-1 derived macrophages, we revealed that combustion particles derived from dung trigger secretion of pro-inflammatory cytokines to a greater extent than those derived from wood and charcoal in the absence of serum. In contrast, wood combustion particles induced a significant decrease in secretion of TNF-α at the highest concentration tested as well as showing a negative linear trend for IL-8 at serum-free conditions. This could indicate immunomodulatory properties but could to some extent be explained by the low-to-moderate cytotoxicity at this concentration (approx. 30% reduction in metabolic activity at 100 µg/mL).

In agreement with the results in THP-1 derived macrophages, moderate pro-inflammatory effects of dung biomass smoke have been reported in primary human small airway epithelial cells exposed at the air–liquid interface, independent of dung origin (McCarthy et al. 2016, 2017). Other studies report no or modest increase of cytokine release in various cell models for biomass particles generated from beech wood in a conventional log wood burner (Krapf et al. 2017) and wood and charcoal in an open fireplace (Marchetti et al. 2019). An in vivo study by Sussan et al. (2014) demonstrated significantly higher inflammatory effects following exposure to PM from cow dung compared to wood, including pro-inflammatory cytokine production, neutrophilic inflammation, airway resistance and hyperresponsiveness under acute conditions. Yet, eosinophilic inflammation, PM-specific antibody responses and alveolar destruction were higher for exposure to wood PM compared to dung PM after sub-chronic exposure. Cytokine induction has further been reported for DEP in vitro (Boland et al. 1999; Mazzarella et al. 2007). The discrepancy in inflammatory response between studies could be explained by the choice of (cell) model, endpoint, method of exposure but also the composition and size of the tested particles. Studies have further suggested biomass exposure to result in the modulation of the immune system resulting in increased susceptibility towards respiratory infections both in vitro (McCarthy et al. 2017) and in vivo (Gibbs-Flournoy et al. 2018; McCarthy et al. 2022). In agreement, epidemiological evidence has demonstrated exposure to biomass smoke to correlate with an increased risk of acute respiratory infections (Gordon et al. 2014).

Interestingly, particles derived from the three types of solid biomass fuels did not cause oxidative stress in BEAS-2B cells under the tested conditions. Previous studies have shown high oxidative potential from particles derived by the combustion of dung (Mudway et al. 2005), wood (Tapanainen et al. 2012; Kurmi et al. 2013) and mixed biomass (Kurmi et al. 2013). The study by Mudway et al. (2005) suggests the oxidative activity of dung biomass particles to be largely attributable to their transition metal content such as Fe and Cu. Yet, Kurmi et al. (2013) demonstrate a negligible contribution by redox active metals in biomass PM. The reference DEP (NIST 2975), while showing an oxidative potential by generating acellular ROS, did not lead to oxidative stress in the BEAS-2B cells. This is in line with the study by Bengalli et al. (2019), reporting no induction of ROS in response to NIST 2975 in BEAS-2B cells. However, NIST 2975 particles have previously been shown to modestly induce ROS in A549 cells (Wu et al. 2022), which underscores the influence of cell model used. The SRM DEP NIST 1650, which is generated from a heavier engine compared to NIST 2975, has been reported to induce ROS in several models in vitro (Bonvallot et al. 2002; Baulig et al. 2003; Jacobsen et al. 2008).

With the inclusion of two different cell lines, we could observe differences in their sensitivity towards combustion particles derived from traditional solid biomass fuels. Our results suggest BEAS-2B to be more sensitive in the cytotoxic and genotoxic endpoints in response to combustion particles, displaying a higher magnitude of effect at lower exposure concentrations compared with what was observed in THP-1 derived macrophages. THP-1 derived macrophages, being more immunocompetent, displayed a higher sensitivity in revealing pro-inflammatory effects of the combustion particles. THP-1 cells are often used as a model for innate immune cells and have been shown to be capable of producing a relevant repertoire of cytokines in response to nanoparticles (Bhattacharya et al. 2017). Important to note is that we did not quantify the cellular dose in either cell model, therefore we can only speculate on the difference in sensitivity based on the applied nominal dose. However, a difference in cellular dose between the cell models cannot be ruled out due to, e.g., differences in phagocytic capacity as well as in the influence of the different cell media on the behavior of the particles (as observed in the DLS data).

THP-1 derived macrophages were exposed to particle suspensions in either serum-enriched or serum-free medium, where wood combustion particles resulted in more elevated cytotoxic effects in serum-free conditions. The higher cytotoxic effects observed in serum-free conditions can likely be explained by greater particle agglomeration and subsequent sedimentation in serum-free medium, as seen in the DLS results, resulting in an increased deposition of particle mass on the cells. We have previously reported similar findings for Ag nanoparticles where particle sedimentation and cytotoxicity were amplified in serum-free conditions compared with serum-enriched conditions in THP-1 derived macrophages (Gliga et al. 2020). Yet, a comparable potency was in general observed for the endpoints of DNA damage and release of inflammatory cytokines in THP-1 derived macrophages irrespective of presence of serum. Collectively, our findings highlight that cell models have varying sensitivity towards specific endpoints and therefore, multiple cell models should be considered in order to get a comprehensive evaluation of the toxicity. In addition, our findings emphasize the potential impact of cell medium composition on the observed effects in vitro.

A recent commentary by Lewis et al. (2023) underscores the need for increased attention towards indoor air pollution. The PM_2.5_ investigated in this study was generated in a laboratory setup yet under realistic conditions relevant for the indoor combustion of solid biomass fuels in sub-Saharan Africa. It is however important to note that most of the particle characterization, including PAH analysis, as well as cell exposure was conducted after collection and extraction of PM. Consequently, the particles investigated in this study might not fully represent the particle characteristics, like PAH composition, of that found during the time of particle generation. In addition, we can only draw conclusions on the toxic effects of the PM generated during the combustion of solid fuels but not the whole biomass exhaust including the gaseous fraction and its related compounds such as low-molecular weight PAHs and other volatile/semi-volatile compounds.

As previously described in Eriksson et al. (2022), the PM_2.5_ mass concentration levels varied greatly depending on the biomass source demonstrating concentrations levels generated from wood to be more than one order of magnitude higher than from charcoal, with the levels generated by dung being yet another order of magnitude higher. The difference in acute toxic responses between dung, eucalyptus wood and charcoal exposure presented in this study may therefore be considered even greater when accounting for the difference in exposure levels. In particular, the combustion of dung results in a considerably higher exposure, approximately 50 times compared to that following the combustion of eucalyptus charcoal during a similar time frame. Combustion of dung, which is generally considered the fuel of last resort used by households of lowest socioeconomic status, results in the highest emissions. The high emission generation rate coupled with the heightened toxic potency of biomass particles derived from dung compared to cleaner alternatives such as charcoal (and our reference DEP), points towards the most resource constrained households being at greatest risk. Nonetheless, the exposure, based on 4 h cooking, is expected to exceed both the annual and 24-h average of the WHO guidelines for healthy air for all three solid biomass fuels investigated in this study (Eriksson et al. 2022). Encouraging the adoption of cleaner fuel alternatives is crucial in reducing hazardous exposures in households across sub-Saharan Africa and in this context, studies like this one can provide insight and input. The influence of alternative stoves to decrease PM concentrations has been shown previously (Ezzati et al. 2000; de la Sota et al. 2018; Gibbs-Flournoy et al. 2018).

## Conclusions

To our knowledge, this is the first in vitro study comparing the toxicity of PM from different solid fuels of relevance for sub-Saharan Africa. Our findings reveal that particles produced through the combustion of these fuels are cytotoxic, genotoxic and trigger inflammatory response in human bronchial epithelial cells and/or monocyte derived macrophages. Notably, particles emitted from the less energy dense biomass fuels such as cow dung and eucalyptus wood were more potent in inducing cytotoxic effects in vitro in epithelial cells. Similarly, dung which is the least energy dense, and cheapest, fuel was the most potent at inducing secretion of pro-inflammatory cytokines in macrophage-like cells. On the other hand, all particles were equally potent at inducing DNA damage in bronchial cells, irrespective of their PAH content. Our study therefore suggests the total PAH content to be associated with cytotoxic effects to a larger extent compared to genotoxicity. Overall, our data highlights that the type of fuel largely influences the toxic profile of the emitted particles. Given that Africa is heavily burdened by air pollution from indoor biomass burning, much remains to be elucidated regarding the role of combustion process and type of fuel for the potential toxicity of the emitted particles. This study emphasizes the need for more research within this field to fully understand the link between physicochemical properties and toxic mechanisms of particles generated from traditional biomass fuels in order to better understand and mitigate health effects related to household air pollution in sub-Saharan Africa.

## Supplementary Information

Below is the link to the electronic supplementary material.

Supplementary file1 (PDF 891 KB)

## Data Availability

The datasets generated during and/or analysed during the current study are available from the corresponding author on reasonable request.
