# Supplementary material for: Toxicity of particles derived from combustion of Ethiopian traditional biomass fuels in human bronchial and macrophage-like cells
Source: Arch Toxicol. 2024 Mar 1;98(5):1515–32. doi: 10.1007/s00204-024-03692-8 (PMC10965653; doi:10.1007/s00204-024-03692-8)
Supplement: Supplementary file 1 — Supplementary file1 (PDF 891 KB) [file 204_2024_3692_MOESM1_ESM.pdf]

Supplementary tables and figures

Toxicity of particles derived from combustion of Ethiopian traditional biomass fuels in human bronchial and macrophage-like cells

Sarah McCarrick[1*], Mathilde N. Delaval[1,2*], Ulrike M. Dauter[1], Annette M. Krais[3], Anastasiia Snigireva[1], Asmamaw Abera[4-6], Karin Broberg[1], Axel C. Eriksson[4-5], Christina Isaxon[4-5] and Anda R. Gliga[1#]

[1]Institute of Environmental Medicine, Karolinska Institutet, Stockholm, Sweden
[2]Joint Mass Spectrometry Centre (JMSC), Cooperation Group Comprehensive Molecular Analytics, Helmholtz Munich, Neuherberg, Germany
[3]Division of Occupational and Environmental Medicine, Department of Laboratory Medicine, Lund University, Lund, Sweden
[4]Ergonomics and Aerosol Technology, Lund University, Lund, Sweden
[5]NanoLund, Lund University, Lund, Sweden
[6]Addis Ababa University, Addis Ababa, Ethiopia

* equal contribution

# corresponding author

Table S1. Measured content of native PAHs and PAH derivatives in solid biomass particles and NIST2975 reference material.

		Dung	Wood	Charcoal	NIST 2975	Molecular weight
		ng/mg particles				
PAHs	naphthalene	0.71	0.41	0.38	1.62	Low (2-3 rings)
	biphenyl	0.19	0.08	0.06	0.38	
	acenaphthylene	0.88	0.35	0.09	0.10	
	acenaphthene	0.15	0.03	0.03	0.01	
	fluorene	2.43	0.56	0.07	0.27	
	phenanthrene	56.62	62.44	1.27	7.93	
	anthracene	11.50	9.66	0.27	0.25	
	fluoranthene	62.72	214.66	19.07	12.21	Middle (4 rings + retene)
	pyrene	59.81	238.39	24.31	2.93	
	retene	0.76	0.45	0.91	1.89	
	benzo(a)anthracene	21.38	240.74	5.18	0.72	High (5-6 rings)
	chrysene	34.29	188.24	7.01	2.62	
	benzo(b)fluoranthene	27.36	505.70	15.46	5.99	
	benzo(k)fluoranthene	7.79	114.85	4.60	0.69	
	benzo(e)pyrene	6.65	95.61	5.36	0.60	
	benzo(a)pyrene	20.68	372.94	7.84	0.72	
	perylene	4.14	82.02	1.93	0.12	
	indeno(1,2,3-c,d)pyrene	13.31	252.27	6.60	0.66	
	dibenzo(a,h)anthracene	1.17	39.97	1.05	0.01	
	benzo(g,h,i)perylene	12.82	256.75	10.74	0.87	
	Coronene	6.23	154.84	2.51	0.70	
Alkyl PAHs	2-methylnaphthalene	0.53	0.17	0.24	0.98	
	1-methylnaphthalene	0.43	0.12	0.15	0.63	
	2,3-dimethylnaphthalene	0.24	0.04	0.06	0.28	
	2,3,5-trimethylnaphthalene	0.03	0.01	0.02	0.05	
	1-methylfluorene	3.77	0.42	0.06	0.25	
	4-methylphenanthrene	12.15	4.03	0.38	0.70	
	3-methylphenanthrene	28.54	7.39	0.59	1.61	
	1-methylphenanthrene	0.15	0.09	0.01	0.01	
	1-methylanthracene	4.71	1.66	0.17	0.19	
	2-phenylnaphthalene	19.28	18.63	1.83	1.17	
	1-methylfluoranthene	6.57	14.35	0.98	0.24	
	1-methylpyrene	13.12	41.93	2.25	0.43	
	2-methylchrysene	9.34	15.45	0.61	0.29	
	5-methylchrysene	0.09	0.38	0.07	0.01	
DBTs	dibenzothiophene	1.29	0.13	0.03	0.13	
	2-methyldibenzothiophene	0.29	0.01	0.04	0.22	
	1-methyldibenzothiophene	2.25	0.32	0.11	0.44	
	4-methyldibenzothiophene	0.16	0.08	0.02	0.02	
	2,8-dimethyldibenzothiophene	0.53	0.18	0.06	0.16	
	2,4,7-trimethyldibenzothiophene	0.09	0.01	0.06	0.06	

Nitro PAHs	1-Nitronaphthalene	0.27	0.33	0.06	0.04
	2-Nitronapthalene	0.28	0.23	0.04	0.03
	1,5 dinitronaphthalene	0.01	0.00	0.05	0.01
	5-nitro acenapthalene	3.53	4.21	0.05	1.18
	2-Nitrofluorene	0.08	0.23	0.07	0.51
	9-Nitroanthracene	0.82	9.44	0.12	1.03
	9-Nitrophenanthrene	0.63	0.93	0.05	0.54
	3-Nitrofluoranthene	1.34	1.97	0.18	2.56
	4-Nitropyrene	0.87	9.28	0.70	2.70
	1-Nitropyrene	0.03	0.12	0.03	5.80
	2-Nitropyrene	0.03	0.25	0.08	18.66
	7-Nitrobenz[a]anthracene	0.06	0.48	0.08	1.76
	6-Nitrochrysene	0.01	0.03	0.04	0.31
	3-Nitrobenzanthrone	0.02	0.09	0.03	0.02
	1,3-Dinitropyrene	0.01	0.04	0.03	0.52
	1,6-Dinitropyrene	0.01	0.00	0.04	1.34
	1,8-Dinitropyrene	0.09	0.04	0.04	2.21
	6-Nitrobenzo[a]pyrene	80.53	15.85	3.89	8.78
	3-Nitroperylene	0.03	0.18	0.06	0.01
	7-Nitro dibenz(a,h)anthracene	0.06	0.02	0.03	0.02
oxy PaHs	Napthalene-1-aldehyde	0.14	0.35	0.05	0.30
	2-Naphthaldehyde	0.11	0.84	0.06	0.41
	p-Fluorenone	20.44	23.88	0.51	3.44
	9,10 Anthraquinone	10.29	23.14	0.72	3.25
	1,4 Anthraquinone	1.86	8.21	0.27	1.06
	Phenanthrene-9-aldehyde	1.57	25.35	0.18	2.82
	Benzo[a]fluorene	11.95	26.64	1.81	0.38
	Benzo[b]fluorene	12.65	27.61	1.82	0.14
	Benzanthrone	12.44	88.79	1.08	0.48
	Benz[a]anthracene-7,12-dione	2.40	13.74	0.80	8.08

Table S2. EC50 concentrations (µg/mL) with 95 % confidence intervals (CI) for the combustion derived particles in BEAS-2B or THP-1 derived macrophages (THP-1*) as per the Alamar Blue (AB) assay or LDH assay.

			**Dung**	**Wood**	**Charcoal**	**DEP**
			µg/mL			
**BEAS-2B**	**AB**	**24h**	35, CI [25-47]	28, CI [22-36]	>150	>150
		**48h**	16, CI [12-21]	11, CI [8-15]	>150	>150
	**LDH**	**24h**	123, CI [78-272]	>150	>150	>150
		**48h**	82, CI [50-187]	88, CI [56-170]	>150	>150
**THP-1***	**AB**	**24h + serum**	>150	>150	>150	>150
		**24 h -serum**	>150	133, CI [115-162]	>150	>150

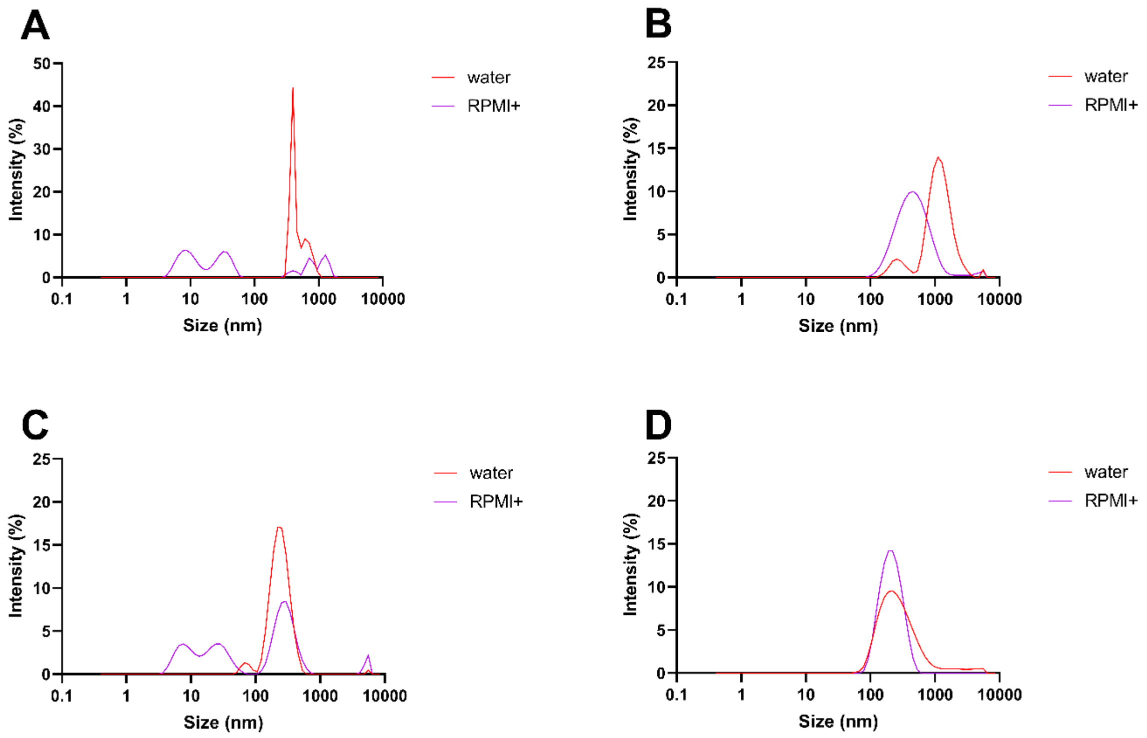

Figure S1. Hydrodynamic size distribution of particles generated from combustion of solid fuel (A – dung, B – eucalyptus wood, C – eucalyptus charcoal) and diesel exhaust particles (D) dispersed (100 µg/mL) in water or serum-containing RPMI (RPMI+). Data is evaluated by dynamic light scattering and expressed as intensity (%).

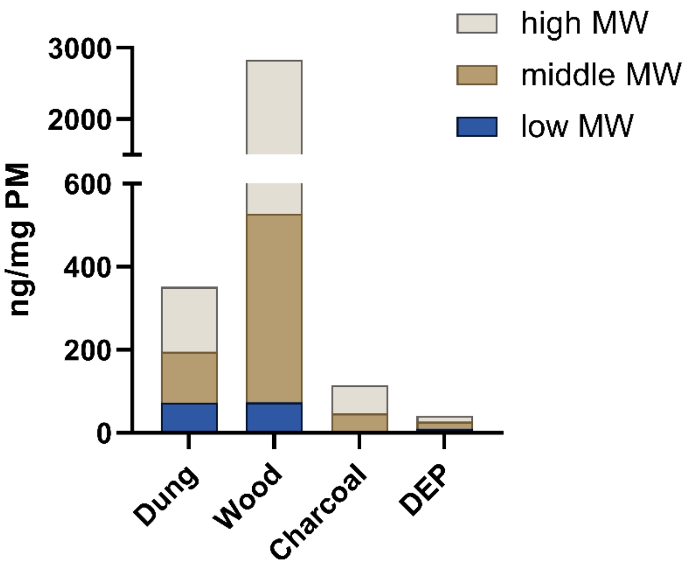

Figure S2. PAH composition of biomass particles generated from solid fuels or diesel exhaust reference particles (NIST 2975) as given in groups of low (2-3 rings), middle (4 rings + retene) and high (5-6 rings) molecular weight PAHs.

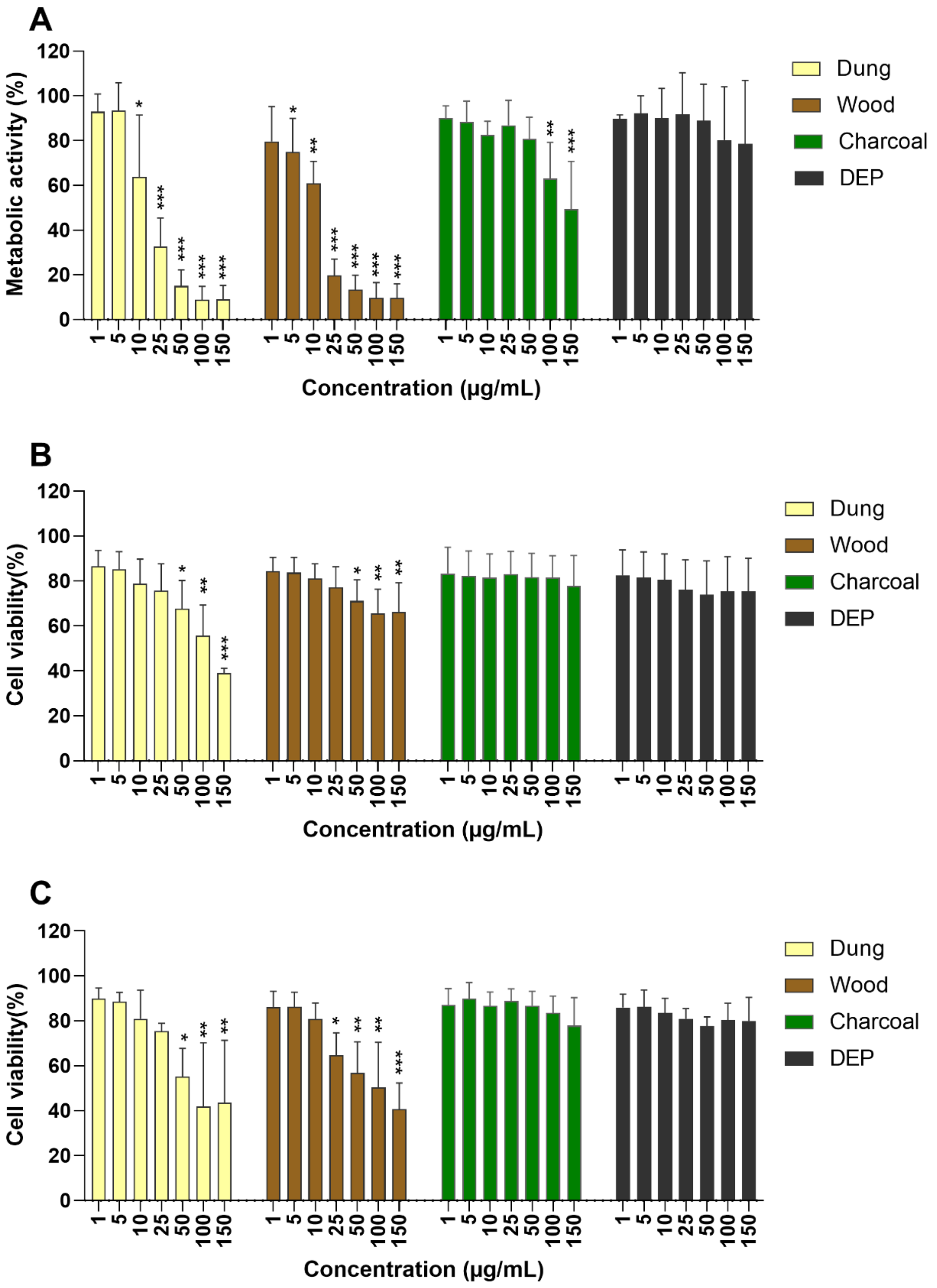

Figure S3. Cytotoxicity of combustion particles in human bronchial cells. BEAS-2B cells were exposed to particles generated from combustion of solid fuel (dung, eucalyptus wood, eucalyptus charcoal) and diesel exhaust particles for 24 h (B) and 48 h (A, C). Cell viability was assessed using Alamar Blue assay (A) and LDH assay (B, C) and results are expressed as % metabolic activity for the Alamar Blue assay and as % cell viability for the LDH assay as compared to the untreated control. Results are presented as mean ± S.D.(n=3). Statistically significant differences, as compared to the control are labelled with asterisks (* for P-value < 0.05, ** for P-value < 0.01, *** for P-value < 0.001).

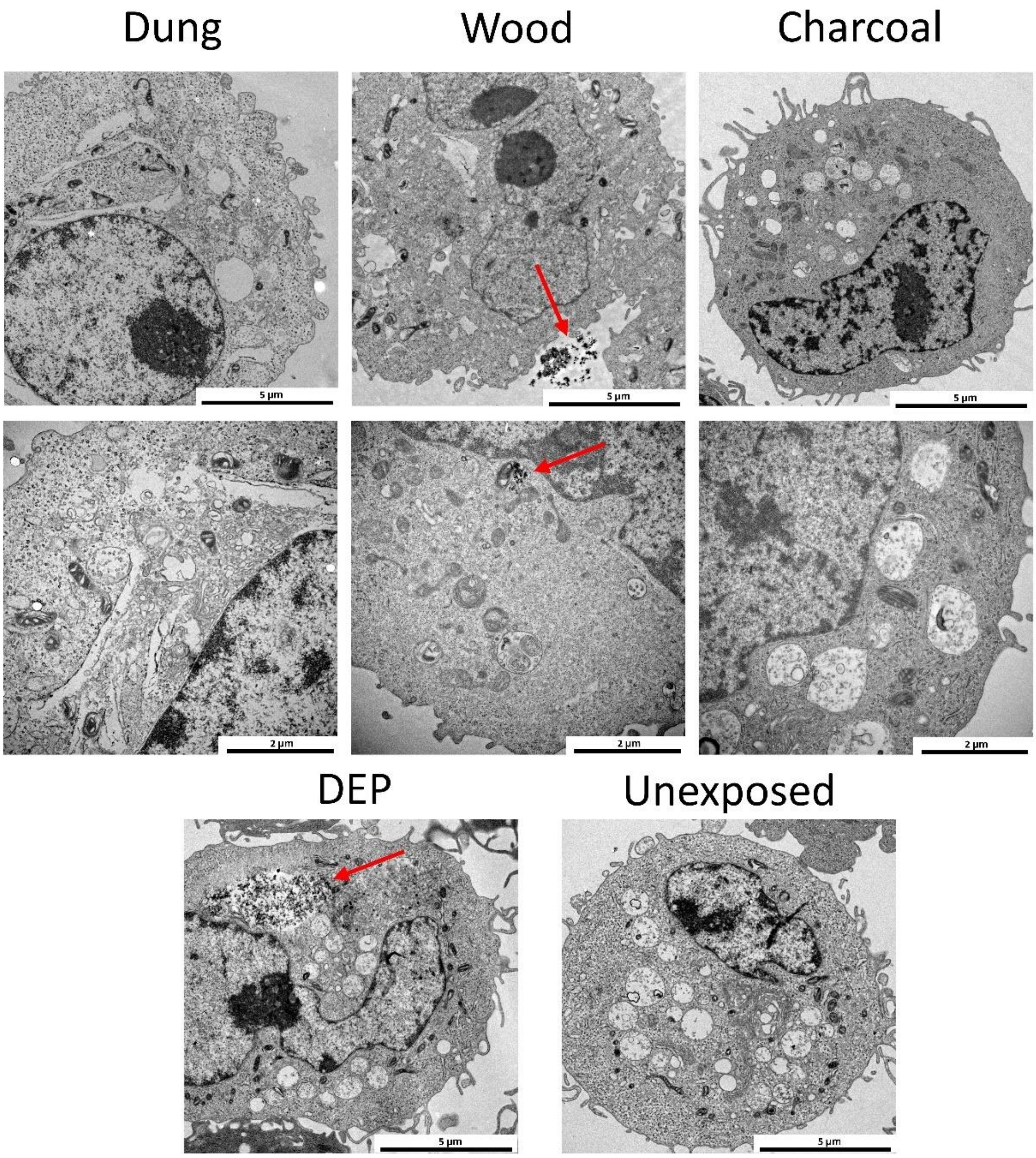

Figure S4. Transmission electron microscopy images of BEAS-2B cells exposed (24 h) to combustion particles including those derived from dung (25 µg/ml), eucalyptus wood (25 µg/ml), eucalyptus charcoal (25 µg/ml) and diesel exhaust particles (5 µg/ml). Red arrows point towards particles.

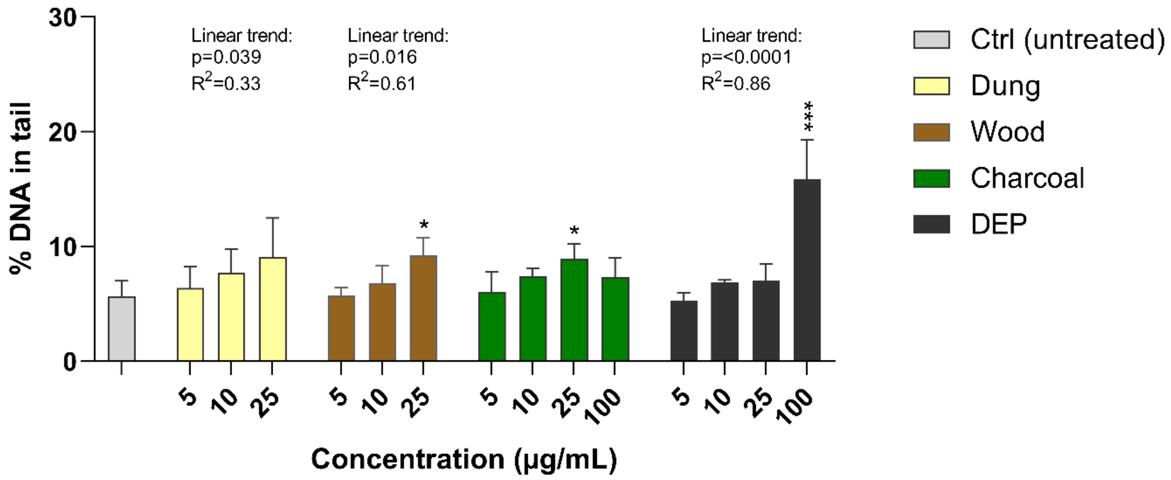

Figure S5. Induction of DNA damage in bronchial epithelial cells exposed to combustion particles for 4 h. DNA strand breaks following exposure to 5, 10, 25 or 100 µg/mL combustion particles at 4 h was quantified by the comet assay under alkaline conditions. DNA damage was quantified as percent of DNA in the comet tail. Results are presented as mean values ± S.D. (n = 3, n = 2 for charcoal and DEP 10 µg/mL). Statistically significant differences, as compared to the control by one-way ANOVA followed by Dunnett's are labelled with asterisks (* for P-value < 0.05, ** for P-value < 0.01, *** for P-value < 0.001). Linear trends were determined by simple linear regression and displayed above each exposure group if significant (p<0.05).

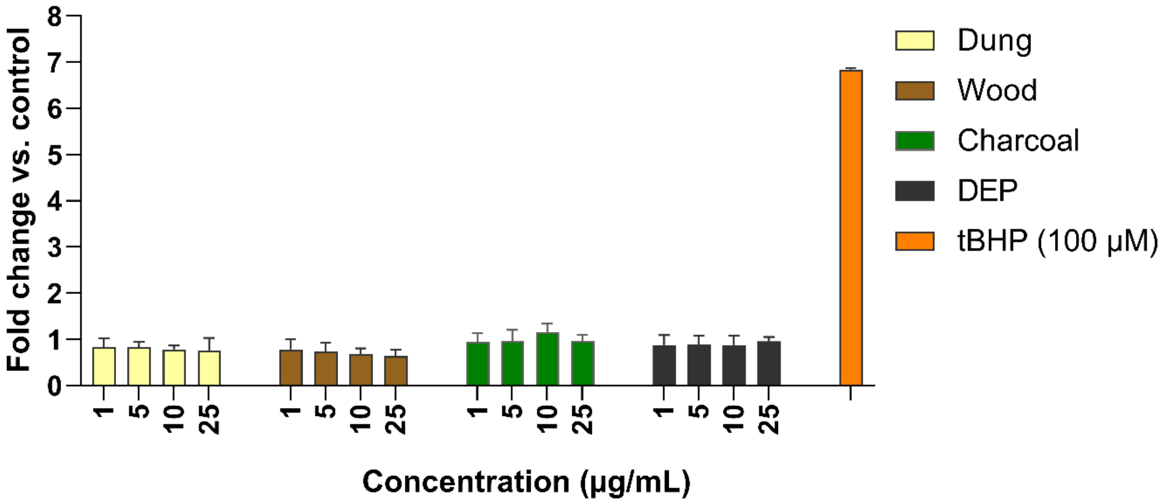

Figure S6. Generation of reactive oxygen species (ROS) in human bronchial cells exposed to combustion particles. BEAS-2B cells were exposed to particles generated from combustion of solid fuel (dung, eucalyptus wood, eucalyptus charcoal) and diesel exhaust particles for 4 h. ROS generation was evaluated using the DCFH-DA assay and results are expressed as fold change versus untreated control. Results are presented as mean ± S.D. (n=3). tBHP (100 μM) was used as a positive control.
